# An Introduction to Diseases of the Nervous System

**Published:** 1900-09

**Authors:** 


**An Introduction to Diseases of the Nervous System.** By H. Campbell Thomson, M.D. Pp. 123. London: Baillière, Tindall and Cox. 1899.

The author says that this little work is intended only as an introduction to the study of diseases of the nervous system; it is therefore written in a simple manner, and deals with a limited part of the subject, covering the ground indicated by his experience in teaching students. After a short sketch of the general structure of the nervous system and of its motor and sensory divisions, the main part of the book is taken up with a description of the actions of the muscles of the body. This description is clear and accurate, and just what a student needs in beginning the study of this subject. He will find this book of much service to him, and may depend on the information it gives him. The author has very wisely, by limiting his scope, avoided all risk of making his work a cram-book. The chapter on the localisation of injuries and diseases of the spinal cord requires to be amplified; it is at present too short to be of real service in what the beginner often finds a difficult subject. Otherwise, we can highly recommend the book, and the illustrations are good.

